# Fecal bacteria-free filtrate transplantation is proved as an effective way for the recovery of radiation-induced individuals in mice

**DOI:** 10.3389/fcimb.2023.1343752

**Published:** 2024-01-31

**Authors:** Hang Zhang, Miaomiao Dong, Jixia Zheng, Yapeng Yang, Jinhui He, Tianhao Liu, Hong Wei

**Affiliations:** ^1^ State Key Laboratory of Agricultural Microbiology, College of Animal Science and Technology, College of Animal Medicine, Huazhong Agricultural University, Wuhan, China; ^2^ Department of Gastroenterology, Affiliated Hospital of Jiangnan University, Wuxi, China

**Keywords:** fecal bacteria-free filtrate transplantation, intestinal mucosal barrier, gut microbiota, radiation damage, metabolism

## Abstract

**Background:**

Ionizing radiation can cause intestinal microecological dysbiosis, resulting in changes in the composition and function of gut microbiota. Altered gut microbiota is closely related to the development and progression of radiation-induced intestinal damage. Although microbiota-oriented therapeutic options such as fecal microbiota transplantation (FMT) have shown some efficacy in treating radiation toxicity, safety concerns endure. Therefore, fecal bacteria-free filtrate transplantation (FFT), which has the potential to become a possible alternative therapy, is well worth investigating. Herein, we performed FFT in a mouse model of radiation exposure and monitored its effects on radiation damage phenotypes, gut microbiota, and metabolomic profiles to assess the effectiveness of FFT as an alternative therapy to FMT safety concerns.

**Results:**

FFT treatment conferred radioprotection against radiation-induced toxicity, representing as better intestinal integrity, robust proinflammatory and anti-inflammatory cytokines homeostasis, and accompanied by significant shifts in gut microbiome. The bacterial compartment of recipients following FFT was characterized by an enrichment of radioprotective microorganisms (members of family *Lachnospiraceae*). Furthermore, metabolome data revealed increased levels of microbially generated short-chain fatty acids (SCFAs) in the feces of FFT mice.

**Conclusions:**

FFT improves radiation-induced intestinal microecological dysbiosis by reshaping intestinal mucosal barrier function, gut microbiota configurations, and host metabolic profiles, highlighting FFT regimen as a promising safe alternative therapy for FMT is effective in the treatment of radiation intestinal injury.

## Introduction

Radiation-induced intestinal injury is a leading complication of radiotherapy for abdominal and pelvic malignancies. A number of recently published literature have claimed that gut microbiota could be disrupted when exposure to ionizing radiation (Cui et al., 2017; Gerassy-Vainberg et al., 2018; Reis Ferreira et al., 2019; Zhang et al., 2022). Radiation enteropathy profoundly affects the quality of life in patients undergoing radiotherapy, however, treatment regimens available remain suboptimal.

The gastrointestinal tract (GI) of mammals is home to a large number of microorganisms, collectively known as gut microbiota, comprising bacteria, fungi, archaea and viruses. It is estimated that the intricate, co-evolved and commensal gut microbiota inhabiting the GI up to 100 trillion microorganisms (Lepage et al., 2013; Guo et al., 2020). As expected, such abundant and diverse microbial communities dictate the heterogeneity of the genes it harbors and their functions, raising the possibility that the crosstalk between microbes and hosts can play a pivotal role in the whole host’s life span (Buford, 2017; Olm et al., 2022). With advances in sequencing technology and improvement of people’s understanding of gut microbiota, increasing number of studies have suggested that the imbalance and/or dysbiosis of gut microecology is closely related to the development and progression of diseases in humans and animals, including type 2 diabetes mellitus (Vals-Delgado et al., 2022), obesity (Alcazar et al., 2022), depressive disorder (Zheng et al., 2016), tumor immunotherapy and radiotherapy (Gopalakrishnan et al., 2018; Cui et al., 2019), and even cognitive decline (Lee et al., 2020).

Probiotics, prebiotics and symbiotic are to date commonly used interventions to modulate gut microbiome in both humans and animals. Recently, fecal microbiota transplantation (FMT), whereby fecal microbiota derived from a healthy donor is transplanted into the recipient’s gut to restore and reconstitute the microbial community compromised by harmful stimuli such as antibiotics usage and ionizing radiation exposure, has been proved to be highly effective in some clinical settings, especially in patients suffering from recurrent *Clostridium difficile* infection (rCDI). Over the past two decades, accumulating evidences have shown that the clinical remission rate of FMT in patients with rCDI up to 90% or higher after single or multi-dose treatments (Bakken et al., 2011; Borody and Khoruts, 2011; Kao et al., 2017).

Although FMT represents a relatively safe bacterial-oriented therapy, safety concern endures because no donor screening standard, however rigorous, can completely rule out the risk of delivering potentially pathogenic microbes from fresh or cryopreserved stool preparations (Wang et al., 2019; Zhang et al., 2020). Of these microbes, many of are inherent to the engraftment of living gut microbiota. Accordingly, further investigations focusing on more safe and efficacious interventions for the therapy of radiation enteropathy and other gut dysbiosis related disorders are warranted. Studies have highlighted that a vast array of viruses residing in the human intestinal tract, which collectively referred as the enteric virome, are considered as an important regulator in shaping gut microbiota via predator-prey dynamics (Shkoporov and Hill, 2019). Of note, the vast majority of enteric virome are bacteriophages, which are specific to infect their bacterial hosts (Mirzaei and Maurice, 2017), indicating of the potential to impact on host pathophysiology. However, the main focus of current research has been on the FMT, whether and how fecal bacteria-free filtrate transplantation (FFT), rather than intact bacterial cells, has any effect on radiation-induced intestinal injury remains poorly understood.

Herein, we postulate that FFT could communicate with bacteriome and modulate the structure and diversity of microbial community, thereby manipulating the gut microbiome and influencing host phenotype after radiotherapy. For the purpose, mice were subjected to total abdominal irradiation (TAI) with a X-ray irradiator to mimic radiation toxicity of radiotherapy in cancer patients. Fecal pellets from both age- and sex-matched donor mice were collected for the preparation of fecal bacteria-free suspension and then introduced into the recipient mice by oral gavage. We assessed the gut microbiota configurations and metabolome profiles of recipient mice and the underlying mechanisms of FFT, to evaluate the efficacy of FFT in radiation-induced intestinal injury.

## Materials and methods

### Animals

**Figure 1:**
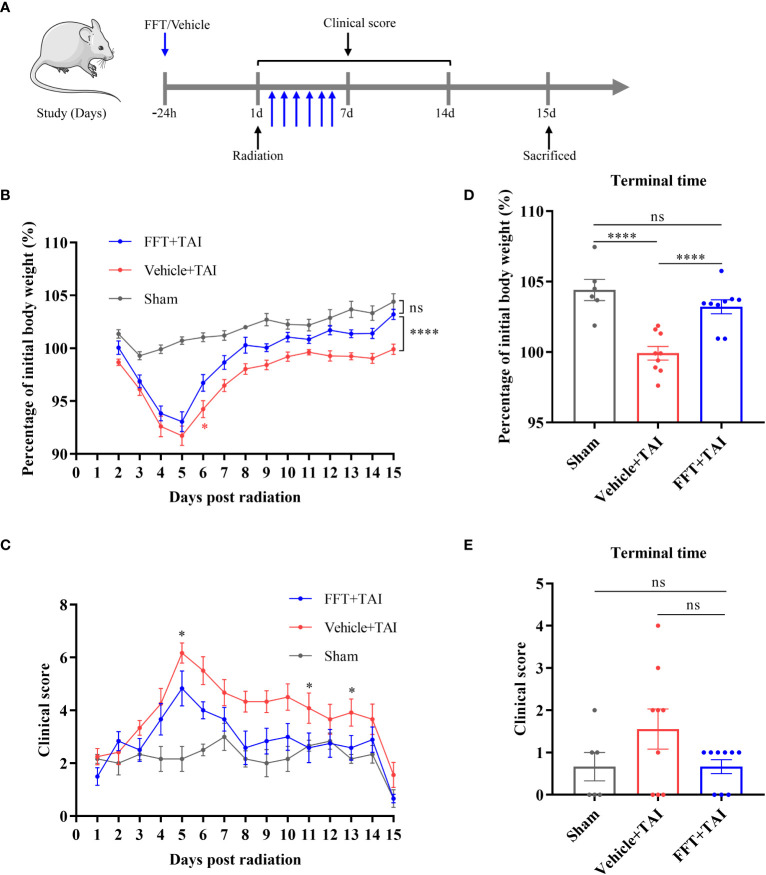
FFT from the health counterparts decreases weight loss and clinical score. **(A)** Illustration of recipient mice with or without radiation challenge received FFT or vehicle treatment at the indicated time points **(B)** Changes in body weight over the course of the experiment. **(C)** Changes in clinical score over the course of the experiment. **(D)** The percentage of initial body weight at the end of the experiment. **(E)** Clinical score at the end of the experiment. FFT, fecal bacteria-free filtrate transplantation; ^*^ *P* < 0.05, ^****^ *P* < 0.0001; ns, no significance. Data are reported as mean ± SEM. Each symbol represents a mouse.

Twenty-four 8- to 10-week-old male C57BL/6 mice weighing approximately 20-22 g were purchased from Hunan SJA laboratory animals Co. Ltd (China), housed in a specific-pathogen-free (SPF) animal facility at the laboratory animal center of Huazhong Agricultural University and fed a standard chow diet ad libitum. All mice used in the present study were acclimatized at least for 5 days before any experimental treatment and maintained under a 12-h light/dark cycle. Before irradiation, the mice were randomly divided into three groups as follows: (1) sham control group, that is, mice were anesthetized by intraperitoneal injection of Avertin (250 mg/kg) without irradiation, (2) vehicle group, where the mice were irradiated under anesthesia using Avertin i.p. and treated with sterile saline, and (3) FFT-treated group, the irradiated mice were gavaged with the FFT preparation at the predetermined time points. The detailed administration schedule is shown in **Figure 1A**. Entire experimental procedures involving mice were performed and approved by the experimental animal welfare ethics committee of Huazhong Agricultural University (license number HZAUMO-2022-0150).

### Irradiated mouse model

A RS-2000-PRO Biological System X-ray irradiator (Rad Source Technologies, Inc., Florida, America) was used in our study. The mice were subjected to a single dose of 10 Gy total abdominal irradiation at a dose rate of 1.053 Gy/min under anesthesia using a special device equipped with the irradiator, so that the whole abdomen was irradiated while the rest of the mouse body was shield by a lead shielding. Following irradiation, the mice were returned to the animal facility, monitored daily, and evaluated for clinical scoring consisting of seven parameters such as weight loss, temperature change, physical appearance, posture, mobility, food consumption, and hydration, as previously described (Guo et al., 2020).

### Preparation of fecal bacteria-free suspension and administration

Fresh stool was collected from 12 gender- and age-matched donor mice from three vendors (four mice from each vendor), and then pooled together to maximize the diversity of viral profile. The fecal bacteria-free suspension was prepared according to a recent study reported by Ott et al. (Ott et al., 2017), with minor modifications. More details are presented in Additional file 1. Mice in FFT-treated group were administered with a 200 microliters aliquot of prepared suspension via oral route on the day before irradiation and daily after irradiation for 7 days. While sham and vehicle groups of mice received an equal volume of sterile vehicle solution as above.

### Measurement of abdominal organs

Fourteen days after irradiation, all 24 recipient mice were euthanized by cervical dislocation, and then the small intestine, spleens, and kidneys were excised and weighted correspondingly. The entire colon segments were also harvested and measured on day 14 following irradiation.

### Quantitative real-time polymerase chain reaction

Cytokine expression was determined by quantitative real-time polymerase chain reaction (qPCR). All experimental mice were sacrificed 14 days post radiation and the colonic segment was harvested. Total RNA was then extracted using FastPure Cell/Tissue Total RNA Isolation kit (Vazyme, Nanjing, China) according to the manufacturer’s instructions. Thereafter, reverse transcription was conducted with HiScript III RT SuperMix for qPCR (+ gDNA wiper) (Vazyme, Nanjing, China) following the manufacturer’s protocols. The qPCR was performed using ChamQ Universal SYBR qPCR Master Mix (Vazyme, Nanjing, China) in a 20 microliters reaction system according to the manufacturer’s guidelines. The primer sets used in this study are listed in Additional file 1. *Gapdh* was used as the internal reference gene.

### Enzyme-linked immunosorbent assay

For preparation of serum samples, peripheral blood was obtained from mouse orbital sinus, centrifuged at 1500 rpm for 15 min at 4°C, snap frozen in liquid nitrogen and stored at -80°C until analysis. The concentrations of target proteins in serum were determined by mouse enzyme-linked immunosorbent assay (ELISA) kits (mlbio, Shanghai, China) in accordance with the standard protocols. Absorbance of each well was measured at 450 nm with a microplate reader.

### Histopathological analysis

Colonic segments were harvested and fixed in 4% paraformaldehyde for at least 24 h at room temperature for histopathological evaluation. For this purpose, the fixed colonic segments were dehydrated, paraffin embedded, chopped into 4-μm sections and stained with hematoxylin and eosin (HE). The slides were evaluated by a GI pathologist blinded to study designs using a semiquantitative radiation injury score (RIS) system consisting of seven histopathology items, as described previously (Langberg et al., 1996; Demirer et al., 2006).

### Immunofluorescence staining

After irradiation at day 14 of 10 Gy TAI, mice were sacrificed under anesthesia. The ileum specimens were harvested, washed in ice-cold phosphate buffer saline (PBS), fixed in 4% paraformaldehyde overnight and chopped into 4-μm sections. Endogenous peroxidase activity was quenched with fluorescence quenching agent for 5 min and rinsed with running water for 10 min. Sections were blocked with bovine serum albumin for 30 min, then incubated with primary antibodies against γH2AX (Abcam, ab81299) overnight at 4°C and washed with PBS. After incubating with secondary antibodies in the dark for 50 min, the slides were subsequently counterstained with 4’, 6-diamidino-2-phenylindole (DAPI). All slides were observed under fluorescence microscope (Eclipse 80i, Nikon) and images were collected.

### Metagenomic sequencing

Fecal pellets were collected from each mouse before sacrificed, snap frozen in liquid nitrogen and stored at -80°C for experimentation. Fecal microbial DNA was extracted using the E.Z.N.A.^®^ stool DNA Kit (Omega Bio-tek, Norcross, GA, USA) according to the manufacturer’s protocols. Then, the DNA obtained was quantified (NanoDrop 2000) followed by library constructed (TruSeq™ DNA Sample Prep Kit, Illumina). The qualified libraries were amplified with a cBot TruSeq PE Cluster Kit (Illumina) and sequenced on an Illumina HiSeq 2000 platform (Truseq SBS Kit v3-HS) with pair-end 150 bp (PE150) mode, with an average sequencing depth of 86, 253,400 reads (13G raw data) per sample. All the raw sequencing data has been deposited into the NCBI public repository with the BioProject ID PRJNA1047338. The raw sequence preprocessing, quality control and optimization and taxonomic annotation are presented in Additional file 1.

### Targeted metabolomics profiling

Mice feces were used for metabolomics analysis. For this purpose, an ultra-performance liquid chromatography coupled to tandem mass spectrometry (UPLC-MS/MS) system (ACQUITY UPLC-Xevo TQ-S, Waters Corp., Milford, MA, USA) was used to quantify metabolites present in feces. Briefly, three to five fecal pellets from each mouse were thawed on ice-bath to diminish degradation, homogenated with zirconium oxide beads and methanol containing internal standard added to extract the metabolites, followed by centrifugation at 18,000 g for 20 min. Subsequent procedures were conducted on the Eppendorf epMotion Workstation (Eppendorf Inc., Humburg, Germany). ACQUITY UPLC BEH C18 1.7 µm VanGuard pre-column (2.1× 5 mm) and ACQUITY UPLC BEH C18 1.7 µm analytical column (2.1 × 100 mm) were employed to determine compounds to be tested. The column temperature was set at 40°C and the flow rate of mobile phase was 0.4 mL/min. The raw data files generated by UPLC-MS/MS were processed using the TMBQ software (v1.0, Metabo-Profile, Shanghai, China), which can perform a collection of data processing, interpretation, and visualization.

### Statistical analysis

All data are reported as mean ± standard error of the mean (mean ± SEM). Statistical differences between two groups were analyzed using either Student T test or the Mann-Whitney U test based on data normality distribution and variance similarity, which were assessed by the Kolmogorov-Smirnov normality test with Dallal-Wilkinson-Lillie for *P* value. Statistical analysis was completed using GraphPad Prism software (version 8.0.1, San Diego, CA, USA). Result with *P* < 0.05 was defined as statistically significant.

## Results

### FFT from the health counterparts decreases weight loss and clinical score

It has been reported that the gut microbiota might be substantially altered by radiation challenge. To determine whether fecal bacteria-free filtrate plays a role in maintaining intestinal homeostasis, we performed FFT in a mouse model to unravel the potential of the sterile fecal filtrate fraction. The radiation exposure significantly reduced body weight in both vehicle- (*P* < 0.0001) and FFT-treated mice within the first 5 days relative to sham-irradiated mice (**Figure 1B**). Intriguingly, FFT treatment clearly erased (*P* < 0.0001) the body weight loss compared with vehicle group of mice (**Figure 1D**), and we also found that there was no statistically significant between the FFT and sham treatment groups at termination of the experiment (**Figure 1D**). Besides that, the clinical score was significantly higher (*P* < 0.05) in vehicle treatment group than in sham control group, whereas orally administered FFT markedly reversed the tendency (**Figures 1C, E**).

### FFT ameliorates radiation caused GI toxicities

**Figure 2:**
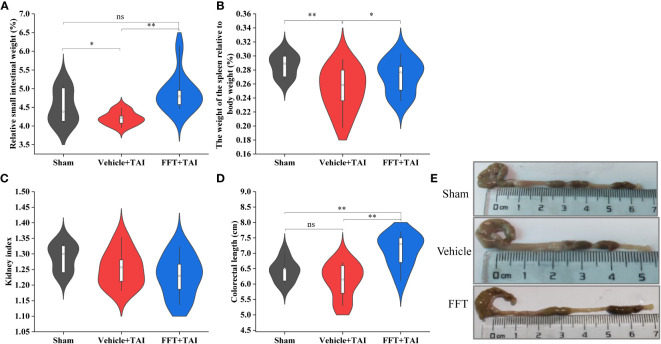
FFT ameliorates GI toxicities caused by radiation exposure. **(A)** Relative weight of the small intestine. **(B)** Relative weight of the spleen. **(C)** Kidney index. **(D)** Colorectal length. **(E)** Representative photo of the colorectal. FFT, fecal bacteria-free filtrate transplantation; ^*^ *P* < 0.05, ^**^ *P* < 0.01; ns, no significance. Data are reported as mean ± SEM.

At termination, the abdominal organs and tissues, including the entire small intestine, colon section, spleens and kidneys, were harvested and measured to identify whether FFT treatment had a radioprotective effect against radiation caused GI injury. As shown in **Figure 2A**, the relative weight of small intestine was overtly decreased (*P* < 0.05) in vehicle treated mice than in sham controls, whereas FFT treatment remarkedly augmented (*P* < 0.01) the relative weight of small intestinal. In parallel, the size and relative weight of the spleens were also significantly reduced (*P* < 0.01) in mice subjected to 10 Gy of TAI challenge compared with sham irradiated mice; nevertheless, FFT treatment slightly abrogated (*P* < 0.05) the changes (**Figure 2B**). The kidney index, which refers to the percentage of wet kidney weight relative to body weight, was decreased slightly in irradiated mice with or without FFT gavage compared with sham control mice, although there was no statistical difference between the two groups (**Figure 2C**). As expected, TAI exposure resulted in shortened colorectal length, but FFT regimen significantly prolonged (*P* < 0.01) colorectal length (**Figures 2D, E**). Taken together, these observations suggest that FFT treatment ameliorates radiation caused GI toxicities.

### FFT restores radiation caused GI structural and functional damage

**Figure 3:**
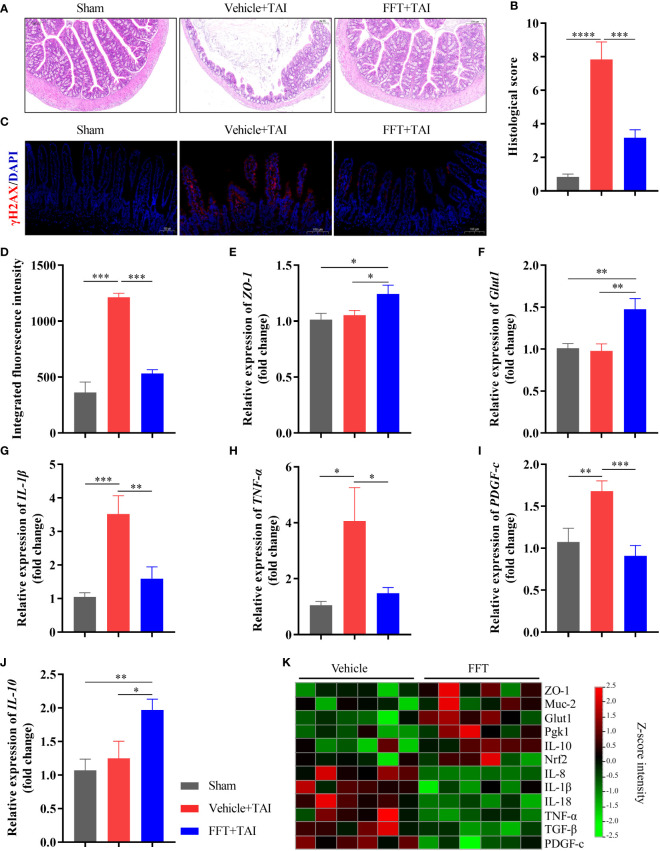
FFT restores radiation caused intestinal structural and functional damage. **(A,B)** HE staining of colonic tissue and histological evaluation. **(C, D)** Immunofluorescence staining of γ-H2AX and fluorescence intensity. **(E)** Relative mRNA expression level of *ZO-1*. **(F)** Relative mRNA expression level of *Glut1*. **(G)** Relative mRNA expression level of *IL-1β*. **(H)** Relative mRNA expression level of *TNF-α*. **(I)** Relative mRNA expression level of *PDGF-C*. **(J)** Relative mRNA expression level of *IL-10*. **(K)** qPCR-based expression array of genes of interest. ^*^ *P* < 0.05, ^**^ *P* < 0.01, ^***^ *P* < 0.001. Data are reported as mean ± SEM.

To further interrogate how FFT treatment protects mice against radiation-induced GI structure and function damage, we first stained the colonic tissues of mice with HE staining to conducted overall histopathological evaluation. Histopathological analysis showed that RIS score of the FFT group was significantly lower (*P* < 0.001) than that of the vehicle group (**Figures 3A, B**), indicating that FFT might exert a protective effect on intestinal injury caused by radiation. Immunofluorescence staining of γ-H2AX, a marker of DNA damage, further validated that FFT treatment significantly reduced (*P* < 0.001) radiation induced DNA damage in intestinal cells compared with vehicle group (**Figures 3C, D**).

To better understand the mechanisms by which FFT treatment protects the intestinal tract from radiation induced dysfunction, colonic tissue was used for determination of mRNA expression using qPCR. And results suggested that FFT treatment significantly enhanced (*P* < 0.05) the intestinal integrity in mice subject to TAI (**Figures 3E, F**; **Figure S1**). In parallel, the relative mRNA expression levels of proinflammatory cytokines, including IL-1β (*P* < 0.01), TNF-α (*P* < 0.05) and IL-8 (*P* < 0.01), were dramatically reduced in FFT group compared with vehicle controls (**Figures 3G, H, K**). Notably, FFT treatment also significantly decreased (*P* < 0.001) the extent of fibrosis and heightened (*P* < 0.05) the anti-inflammatory cytokine expression (**Figures 3I, J**). Together, these findings indicate that FFT treatment contributes to the recovery of intestinal integrity and function following radiation exposure.

### FFT ameliorates systemic inflammatory response after radiation exposure

**Figure 4:**
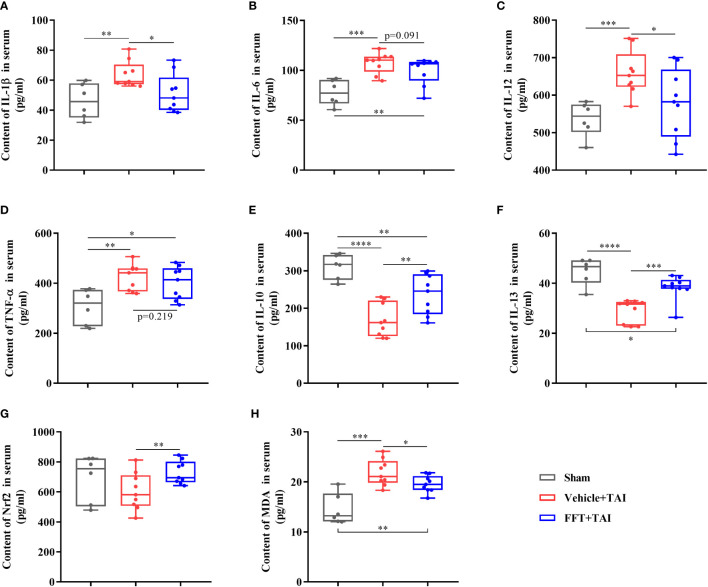
The contents of cytokines in serum. **(A)** Content of IL-1β in serum. **(B)** Content of IL-6 in serum. **(C)** Content of IL-12 in serum. **(D)** Content of TNF-α in serum. **(E)** Content of IL-10 in serum. **(F)** Content of IL-13 in serum. **(G)** Content of Nrf2 in serum. **(H)** Content of MDA in serum. ^*^ *P* < 0.05, ^**^ *P* < 0.01, ^***^ *P* < 0.001, ^****^ *P* < 0.0001. Data are reported as mean ± SEM. Each symbol represents a mouse.

As previously stated, it is obvious that FFT may successfully alleviate radiation induced local intestinal dysfunction by maintaining intestinal integrity and cytokines expression equilibrium, however it is uncertain if FFT has an influence on systemic inflammatory response. Accordingly, we determined the levels of proteins involved in proinflammatory and/or anti-inflammatory response in serum samples using ELISA kits. Consistent with the qPCR results described above, the protein contents of proinflammatory cytokines in serum such as IL-1β (*P* < 0.01), IL-6 (*P* < 0.001), IL-12 (*P* < 0.001) and TNF-α (*P* < 0.01), were dramatically higher in mice only receiving radiation than those in sham controls, but lower in FFT treatment group compared with mice receiving radiation only (**Figures 4A-D**). Furthermore, FFT treatment significantly elevated (*P* < 0.01) the protein contents of anti-inflammatory cytokines in serum compared with vehicle group (**Figures 4E, F**). Radiation challenge can result in the activation of Nrf2 and an increase in MDA (Lu et al., 2019). Additionally, we saw that the levels of Nrf2 (*P* < 0.01) and MDA (*P* < 0.05), which have become markers of the formation and removal of reactive oxygen species, were significantly greater in the FFT treatment group compared to the vehicle group (**Figures 4G, H**). Collectively, our results obtained demonstrate that FFT plays a role in regulating radiation-challenged systemic inflammatory response.

### Alterations in fecal microbiome composition

**Figure 5:**
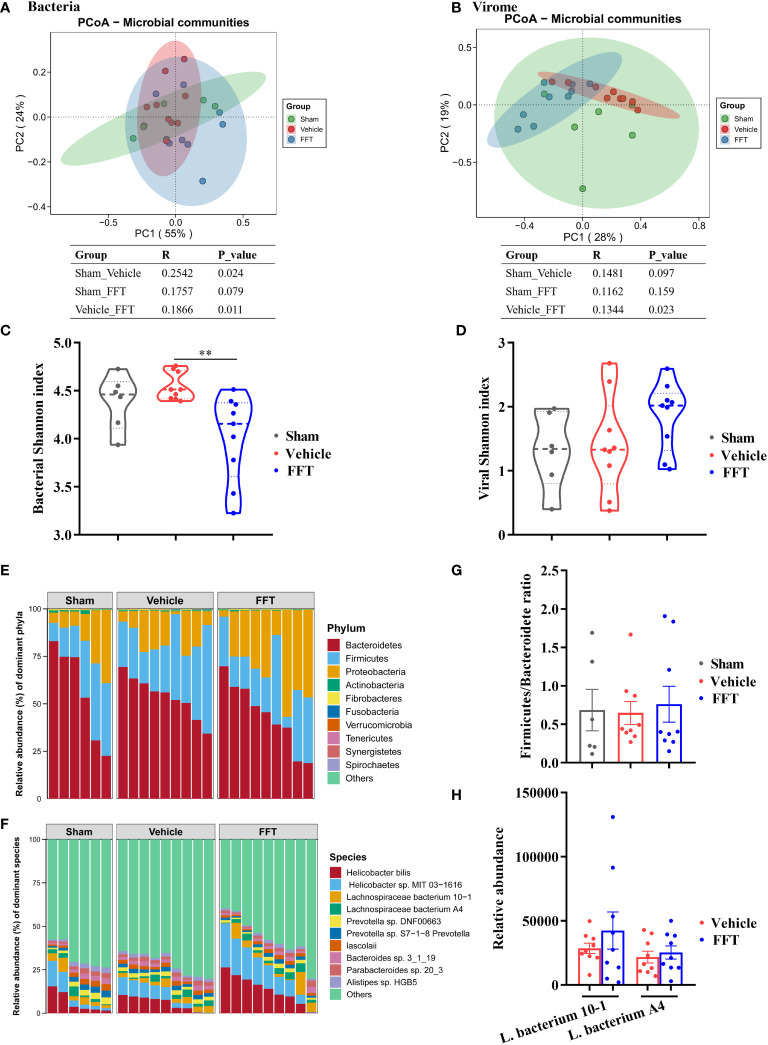
Altered gut microbiome in feces of recipient mice. **(A)** Bacterial principal coordinate analysis (PCoA). **(B)** Shannon index of bacterial community. **(C)** Viral PCoA. **(D)** Shannon index of viral community. **(E)** Fecal bacterial communities at the phylum level. **(F)** Fecal bacterial communities at the species level. **(G)** Ratio of Firmicutes to Bacteroidetes. **(H)** Relative abundance of *Lachnospiraceae* at species level. ^**^ *P* < 0.01. Data in tables represent analysis of similarities (Anosim) based on the Bray-Curtis dissimilarity metric. Data are reported as mean ± SEM. Each symbol represents a mouse.

We analyzed the structure and composition of fecal microbiome at termination, with emphasis on the effect of FFT treatment on fecal bacterial composition. In general, interventional effects were seen in both fecal bacterial and viral compositions. Specifically, the fecal bacterial composition changed significantly (*P* < 0.05) in vehicle group mice relative to sham controls, and the bacterial composition of FFT group mice was likewise different from (*P* < 0.05) the vehicle group mice (**Figure 5A**). Interestingly, the difference between the FFT and the sham group in gut microbiome was not significant (p = 0.079, **Figure 5A**). Changes in fecal virome component across groups were similar to that of the bacteria. Results of analysis of similarities (Anosim) of the Bray-Curtis dissimilarity metrics showed that the fecal viral communities in the FFT group were significantly (*P* < 0.05) different from those in the vehicle group, whereas no significant differences were found in the viral communities of the sham compared with the vehicle group and the sham with the FFT group (**Figure 5B**). There was no significant difference in bacterial Shannon index between the FFT group and the sham group, but interestingly, the bacterial Shannon index of the FFT group was significantly lower than that of the vehicle group; however, FFT treatment did not affect the Shannon index of the viral communities (**Figures 5C, D**).

To further untangle the effect of FFT treatment on bacterial composition, fecal bacterial communities were analyzed at phylum and species levels. We observed that the Bacteroidetes, Firmicutes and Proteobacteria predominated in the gut (**Figure 5E**). In addition, compared with the sham controls, the ratio of Firmicutes to Bacteroidetes decreased in the vehicle group; nevertheless, FFT treatment abrogated the reduction of Firmicutes to Bacteroidetes ratio (**Figure 5G**), although this change did not reach a statistically significant difference between the FFT and vehicle groups. At the species level, the relative abundances of *Helicobacter bilis*, *Helicobacter* sp. *MIT 03-1616*, *Lachnospiraceae bacterium 10-1*, and *Lachnospiraceae bacterium A4* in the FFT group were higher relative to the vehicle group (**Figures 5F, H**). The members of family *Lachnospiraceae* have showed a radioprotective role following total body radiation [6]. Taken together, it was showed that FFT treatment protects mice against radiation caused local and systemic toxicities, which was related to gut microbes.

### Alterations in fecal metabolome profiles

**Figure 6:**
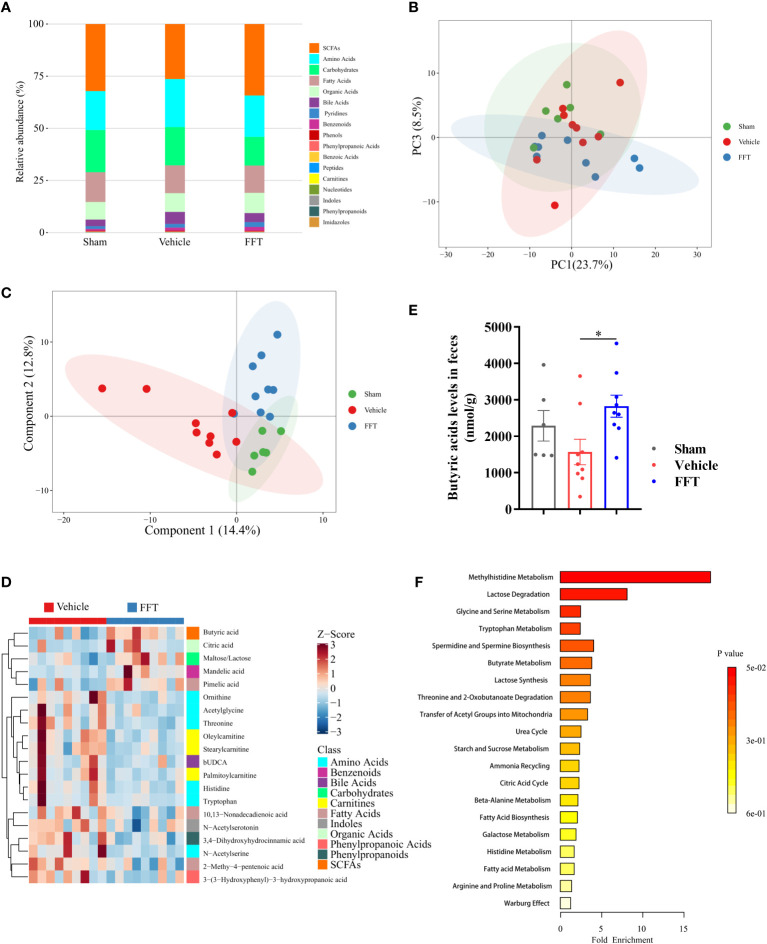
Fecal metabolome profiling of recipient mice. **(A)** A bar chart of the metabolite classification overview. **(B)** PCA plot of feces from sham, vehicle and FFT groups. **(C)** PLS-DA plot of feces from sham, vehicle and FFT groups. **(D)** Heatmap of potential biomarkers between the FFT and vehicle groups, with the screening criteria of *P* < 0.05, |log2FC| >= 0 in univariate analysis and VIP > 1 in multidimensional analysis. **(E)** Butyric acid levels in the feces of mice treated with FFT or vehicle. **(F)** metabolite sets enrichment overview. Data are reported as mean ± SEM. Each symbol represents a mouse. *P < 0.05.

To gain insight into the host-gut microbiota interactions, we performed UPLC-MS/MS metabolomics analysis on feces from mice with or without FFT intervention. Overall, compared with the vehicle group, the metabolic profiles of mice in the FFT group were more similar to that of the sham group. Notably, the relative abundance of short-chain fatty acids (SCFAs), which have showed well radioprotection benefits, was reduced following radiation exposure, while FFT treatment clearly enhanced the relative abundance of SCFAs (**Figure 6A**; **Figure S2**). Further, based on the supervised and unsupervised classification discrimination models, namely principal component analysis (PCA) and partial least-squares discrimination analysis (PLS-DA), we found that the metabolic profiles of FFT group were positioned between the sham and vehicle group (**Figures 6B, C**), supporting the benefit of FFT treatment for the side effects of radiotherapy.

Through the intersection of univariate statistics and multidimensional statistics, 20 significantly altered differential metabolites that more likely to become potential biomarkers were identified between the FFT group and the vehicle group. As shown in **Figure 6D**, the differential metabolites investigated were mainly composed of SCFAs, amino acids, carbohydrates and fatty acids. Of these, a significantly increased level of butyric acids in the FFT group was noted (**Figure 6E**). More specifically, FFT treatment overtly elevated the levels of butyric acid, citric acid, maltose/lactose and pimelic acid compared with the vehicle group, whereas the levels of amino acids metabolites (e.g., ornithine, acetylglycine, threonine and N- acetylserine), carnitines metabolites (e.g., oleylcarnitine and stearylcarnitine), and fatty acids metabolites (e.g., 10, 13-nonadecadienoic acid and 2-methy-4-pentenoic acid), were all markedly decreased. The differential metabolites enriched in the FFT group mainly clustered in the butyrate metabolism pathway and lactose degradation pathway, with an approximately 4-fold change in butyric acid level and an 8-fold change in lactose level (**Figure 6F**). Taken together, our findings suggest that FFT treatment contributes to the production of gut microbiota-derived radioprotective metabolites.

## Discussion

FFT has been successfully used in human beings and animal models for several diseases, including rCDI, necrotizing enterocolitis, type 2 diabetes and obesity (Ott et al., 2017; Rasmussen et al., 2020; Brunse et al., 2022). In the present study, our observations showed that FFT preparation originating from healthy donor mice apparently ameliorated the detrimental effects of radiotherapy in recipient mice, manifested as better body weight gain, lower clinical and histopathological score, attenuated GI toxicity, as well as more robust local and systemic cytokines homeostasis. Furthermore, we also observed that FFT treatment significantly affected and reshaped gut microbiome of the recipients to a large extent. Additionally, the metabolic data indicated that FFT exerted a positive effect on the metabolism of recipient mice, representing as the enrichment of putative radioprotective agents such as SCFAs.

Weight loss has been proposed as a common side effect of radiotherapy for tumors (Xiao et al., 2020). Indeed, we observed here that FFT treatment apparently counteracted the decrease in body weight in mice receiving radiation, whereas no similar effect was found in vehicle treated mice. In parallel, the FFT treatment appeared with a lower clinical sore, which is a reliable measure to assess the severity of radiation induced sickness (Guo et al., 2020). The gut is sensitive to radiation exposure and is susceptible to radiation injury due to the rapid renewal of intestinal epithelial cells. Accordingly, the level of γ-H2AX, a marker intertwined with DNA damage (Lu et al., 2019), was detected in small intestine tissue by immunofluorescence assay. And we demonstrated that FFT treatment significantly mitigated radiation induced DNA damage in intestinal cells compared with vehicle group. Here we tested the role of FFT treatment in ameliorating radiation caused GI toxicities. As expected, FFT administration significantly offset the damage to multiple organs and tissues in the abdomen caused by radiation exposure, as indicated by longer colon length, increased relative weight of the small intestine and spleen. Similar results have also seen in cognate FMT administration (Cui et al., 2017; Xiao et al., 2020).

Radiation exposure can disrupt the homeostasis of the intestinal microenvironment, leading to radiation and inflammatory susceptibility transmission (Gerassy-Vainberg et al., 2018). In the current study, genes expression involved in intestinal integrity and function, and protein level regarding to inflammatory response were detected to analyze the effects of FFT on local and systemic inflammatory response. The results showed that several genes involved in maintaining intestinal integrity and function were differentially expressed between the FFT and vehicle groups, and thereby confirm the beneficial effect of FFT on radiation damage. The *ZO-1* gene plays a pivotal role in the maintenance of the intestinal physical barrier (Wu et al., 2022). The mRNA relative expression level of *ZO-1* in FFT group was significantly higher than that in vehicle group. *Glut1* is an important glucose transporter, and is involved in a list of physiological functions *in vivo*, including maintenance of local and systemic energy homeostasis (He et al., 2022). We observed that radiation challenge lowered the expression of *Glut1* gene, while FFT treatment significantly increased the expression of *Glut1* gene, which may indicate that FFT treatment facilitates glucose transport in the gut, thereby leading to better weight recovery. *Il-1β* and *Tnf-α* are two important mediators of radiation damage, and administration of IL-1β receptor antagonists has been shown to attenuate intestinal tissue damage (Gerassy-Vainberg et al., 2018). PDGF-C acts as an important regulator of radiation proctopathy disease by promoting inflammation and fibrosis in colorectal tissue. Evidence has shown that the relative mRNA expression of *Pdgf-c* is significantly increased in both irradiated mouse model and patients undergoing radiotherapy, and that radiation damage is diminished in *Pdgf-c*-deficient mice (Lu et al., 2021). In the present study, compared with the sham group, the relative expression levels of *Il-1β*, *Tnf-α* and *Pdgf-c* genes were all significantly upregulated in the vehicle group, but comparable between the sham group and the FFT group. Notably, the relative mRNA expression of anti-inflammatory cytokine *Il-10* was significantly increased in the FFT group in comparison with both the sham group and the vehicle group. From the prospect of protein, the trend of changes in proteins of interest in serum across groups was almost consistent with those of the qPCR tests. Overall, our results strongly indicate that FFT treatment positively influences local and systemic inflammatory responses, and to some extent counteracts some of the radiation induced toxicity.

Accumulating evidence demonstrates that radiation exposure disrupts normal gut microbiota composition and structure, and that “deteriorative” gut microbiota is involved in the initiation and progression of radiation injury (Gerassy-Vainberg et al., 2018; Wang et al., 2023). As expected, the FFT treatment significantly affected both the bacterial and viral composition in the gut of recipient mice, characterized by a clear separation of the vehicle group compared to the FFT group, which is in accordance with the findings of a previous study (Rasmussen et al., 2020). A small case report suggested that the diversity of gut virome was associated with clinical remission, whereby the FFT was successfully implemented in patients with CDI (Brunse et al., 2022). In this study, FFT moderately increased viral Shannon index compared with the vehicle group. Contrarily, the bacterial Shannon index of the FFT group was lower than that of the vehicle group, which may be mediated by the predator-prey model (Shkoporov and Hill, 2019). Whether this dichotomy has any unique role in clinical practice remains to be further explored.

It has been shown that the Firmicutes/Bacteroidetes ratio can be used to reflect the homeostasis of gut microbiota (Pan et al., 2022). We found herein that the FFT reversed the decrease in the Firmicutes/Bacteroidetes ratio in irradiated mice towards the gut microbiota composition of the sham group. Furthermore, the differentially enriched microbes in the FFT group were mainly members of family *Lachnospiraceae* and *Helicobacter bilis*, *Helicobacter* sp. *MIT 03-1616*. The members of family *Lachnospiraceae* have been shown to prevent radiation-induced injury in mice (Guo et al., 2020), but whether these two enriched microbial taxa have any radioprotective effect needs to be further investigated. It has been widely accepted that there is a strong correlation between the gut microbiota and the metabolism of its host. In the present study, the fecal metabolome profile of the FFT group was significantly different from the vehicle group and was positioned between those of mice in the sham group and the vehicle group. Moreover, SCFAs were remarkedly enriched when comparing the FFT group with the vehicle group and the sham group with the vehicle group, a confirmation of radioprotection by SCFAs to a certain extent (Li et al., 2020). In addition, the butyrate metabolism was one of the most enriched metabolism pathways of differential metabolites in the FFT group, which was in line with aforementioned findings.

## Conclusion

In conclusion, the results of our study demonstrate radiation-challenged induced alterations in gut microbiota configuration and host metabolism. To the best of our knowledge, we are the first to describe the potential of FFT manipulation to attenuate the adverse effects imparted by radiation exposure. This study highlights that FFT treatment may be a promising route for radiation damage.

## Data availability statement

The data presented in the study are deposited in the NCBI repository, accession number PRJNA1047338.

## Ethics statement

The animal study was approved by the experimental animal welfare ethics committee of Huazhong Agricultural University. The study was conducted in accordance with the local legislation and institutional requirements.

## Author contributions

HZ: Writing – original draft, Conceptualization, Data curation, Formal Analysis, Investigation, Methodology. MD: Investigation, Writing – original draft. JZ: Data curation, Investigation, Writing – original draft. YY: Methodology, Project administration, Writing – original draft. JH: Data curation, Formal analysis, Writing – original draft. TL: Supervision, Writing – review & editing. HW: Conceptualization, Project administration, Resources, Supervision, Validation, Writing – review & editing, Funding acquisition.

## References

[B1] Alcazar M. Escribano J. Ferre N. Closa-Monasterolo R. Selma-Royo M. Feliu A.. (2022). Gut microbiota is associated with metabolic health in children with obesity. Clin. Nutr. 41, 1680–1688. doi: 10.1016/j.clnu.2022.06.007 35777107

[B2] Bakken J. S. Borody T. Brandt L. J. Brill J. V. Demarco D. C. Franzos M. A.. (2011). Treating Clostridium difficile infection with fecal microbiota transplantation. Clin. Gastroenterol. Hepatol. 9, 1044–1049. doi: 10.1016/j.cgh.2011.08.014 21871249 PMC3223289

[B3] Borody T. J. Khoruts A. (2011). Fecal microbiota transplantation and emerging applications. Nat. Rev. Gastroenterol. Hepatol. 9, 88–96. doi: 10.1038/nrgastro.2011.244 22183182

[B4] Brunse A. Deng L. Pan X. Hui Y. Castro-Mejía J. Kot W.. (2022). Fecal filtrate transplantation protects against necrotizing enterocolitis. ISME. J. 16, 686–694. doi: 10.1038/s41396-021-01107-5 34552194 PMC8857206

[B5] Buford T. W. (2017). (Dis)Trust your gut: the gut microbiome in age-related inflammation, health, and disease. Microbiome 5, 80. doi: 10.1186/s40168-017-0296-0 28709450 PMC5512975

[B6] Cui M. Xiao H. Li Y. Zhang S. Dong J. Wang B.. (2019). Sexual dimorphism of gut microbiota dictates therapeutics efficacy of radiation injuries. Adv. Sci. 6, 1901048. doi: 10.1002/advs.201901048 PMC6839645 31728280

[B7] Cui M. Xiao H. Li Y. Zhou L. Zhao S. Luo D.. (2017). Faecal microbiota transplantation protects against radiation-induced toxicity. EMBO Mol. Med. 9, 448–461. doi: 10.15252/emmm.201606932 28242755 PMC5376756

[B8] Demirer S. Aydintug S. Aslim B. Kepenekci I. Sengul N. Evirgen O.. (2006). Effects of probiotics on radiation-induced intestinal injury in rats. Nutrition 22, 179–186. doi: 10.1016/j.nut.2005.08.003 16459231

[B9] Gerassy-Vainberg S. Blatt A. Danin-Poleg Y. Gershovich K. Sabo E. Nevelsky A.. (2018). Radiation induces proinflammatory dysbiosis: transmission of inflammatory susceptibility by host cytokine induction. Gut 67, 97–107. doi: 10.1136/gutjnl-2017-313789 28438965

[B10] Gopalakrishnan V. Helmink B. A. Spencer C. N. Reuben A. Wargo J. A. (2018). The influence of the gut microbiome on cancer, immunity, and cancer immunotherapy. Cancer Cell. 33, 570–580. doi: 10.1016/j.ccell.2018.03.015 29634945 PMC6529202

[B11] Guo H. Chou W. C. Lai Y. Liang K. Tam J. W. Brickey W. J.. (2020). Multi-omics analyses of radiation survivors identify radioprotective microbes and metabolites. Science 370. doi: 10.1126/science.aay9097 PMC7898465 33122357

[B12] He Q. Zhang Y. Ma D. Zhang W. Zhang H. (2022). Lactobacillus casei Zhang exerts anti-obesity effect to obese glut1 and gut-specific-glut1 knockout mice via gut microbiota modulation mediated different metagenomic pathways. Eur. J. Nutr. 61, 2003–2014. doi: 10.1007/s00394-021-02764-0 34984487

[B13] Kao D. Roach B. Silva M. Beck P. Rioux K. Kaplan G. G.. (2017). Effect of Oral Capsule- vs Colonoscopy-Delivered Fecal Microbiota Transplantation on Recurrent Clostridium difficile Infection: A Randomized Clinical Trial. JAMA 318, 1985–1993. doi: 10.1001/jama.2017.17077 29183074 PMC5820695

[B14] Langberg C. W. Sauer T. Reitan J. B. Hauer-Jensen M. (1996). Relationship between intestinal fibrosis and histopathologic and morphometric changes in consequential and late radiation enteropathy. Acta Oncol. 35, 81–87. doi: 10.3109/02841869609098484 8619945

[B15] Lee J. Venna V. R. Durgan D. J. Shi H. Hudobenko J. Putluri N.. (2020). Young versus aged microbiota transplants to germ-free mice: increased short-chain fatty acids and improved cognitive performance. Gut. Microbes 12, 1–14. doi: 10.1080/19490976.2020.1814107 PMC7757789 32897773

[B16] Lepage P. Leclerc M. C. Joossens M. Mondot S. Blottiere H. M. Raes J.. (2013). A metagenomic insight into our gut’s microbiome. Gut 62, 146–158. doi: 10.1136/gutjnl-2011-301805 22525886

[B17] Li Y. Dong J. Xiao H. Zhang S. Wang B. Cui M.. (2020). Gut commensal derived-valeric acid protects against radiation injuries. Gut. Microbes 11, 789–806. doi: 10.1080/19490976.2019.1709387 31931652 PMC7524389

[B18] Lu L. Jiang M. Zhu C. He J. Fan S. (2019). Amelioration of whole abdominal irradiation-induced intestinal injury in mice with 3,3’-Diindolylmethane (DIM). Free Radic. Biol. Med. 130, 244–255. doi: 10.1016/j.freeradbiomed.2018.10.410 30352304

[B19] Lu W. Xie Y. Huang B. Ma T. Wang H. Deng B.. (2021). Platelet-derived growth factor C signaling is a potential therapeutic target for radiation proctopathy. Sci. Transl. Med. 13. doi: 10.1126/scitranslmed.abc2344 33627485

[B20] Mirzaei M. K. Maurice C. F. (2017). Menage a trois in the human gut: interactions between host, bacteria and phages. Nat. Rev. Microbiol. 15, 397–408. doi: 10.1038/nrmicro.2017.30 28461690

[B21] Olm M. R. Dahan D. Carter M. M. Merrill B. D. Yu F. B. Jain S.. (2022). Robust variation in infant gut microbiome assembly across a spectrum of lifestyles. Science 376, 1220–1223. doi: 10.1126/science.abj2972 35679413 PMC9894631

[B22] Ott S. J. Waetzig G. H. Rehman A. Moltzau-Anderson J. Bharti R. Grasis J. A.. (2017). Efficacy of sterile fecal filtrate transfer for treating patients with clostridium difficile infection. Gastroenterology 152, 799–811. doi: 10.1053/j.gastro.2016.11.010 27866880

[B23] Pan Z. Hu Y. Huang Z. Han N. Li Y. Zhuang X.. (2022). Alterations in gut microbiota and metabolites associated with altitude-induced cardiac hypertrophy in rats during hypobaric hypoxia challenge. Sci. China Life Sci. 65, 2093–2113. doi: 10.1007/s11427-021-2056-1 35301705

[B24] Rasmussen T. S. Mentzel C. M. J. Kot W. Castro-Mejia J. L. Zuffa S. Swann J. R.. (2020). Faecal virome transplantation decreases symptoms of type 2 diabetes and obesity in a murine model. Gut 69, 2122–2230. doi: 10.1136/gutjnl-2019-320005 32165408

[B25] Reis Ferreira M. Andreyev H. J. N. Mohammed K. Truelove L. Gowan S. M. Li J.. (2019). Microbiota- and radiotherapy-induced gastrointestinal side-effects (MARS) study: A large pilot study of the microbiome in acute and late-radiation enteropathy. Clin. Cancer Res. 25, 6487–6500. doi: 10.1158/1078-0432.CCR-19-0960 31345839

[B26] Shkoporov A. N. Hill C. (2019). Bacteriophages of the human gut: the “Known unknown” of the microbiome. Cell Host Microbe 25, 195–209. doi: 10.1016/j.chom.2019.01.017 30763534

[B27] Vals-Delgado C. Alcala-Diaz J. F. Molina-Abril H. Roncero-Ramos I. Caspers M. P. M. Schuren F. H. J.. (2022). An altered microbiota pattern precedes Type 2 diabetes mellitus development: From the CORDIOPREV study. J. Adv. Res. 35, 99–108. doi: 10.1016/j.jare.2021.05.001 35024196 PMC8721255

[B28] Wang J. W. Kuo C. H. Kuo F. C. Wang Y. K. Hsu W. H. Yu F. J.. (2019). Fecal microbiota transplantation: Review and update. J. Formos. Med. Assoc. 118, 23–31. doi: 10.1016/j.jfma.2018.08.011 30181015

[B29] Wang Z. Xiao H. Dong J. Li Y. Wang B. Chen Z.. (2023). Sexual dimorphism in gut microbiota dictates therapeutic efficacy of intravenous immunoglobulin on radiotherapy complications. J. Adv. Res. 46, 123–133. doi: 10.1016/j.jare.2022.06.002 35700918 PMC10105085

[B30] Wu Y. Jha R. Li A. Liu H. Zhang Z. Zhang C.. (2022). Probiotics (Lactobacillus plantarum HNU082) supplementation relieves ulcerative colitis by affecting intestinal barrier functions, immunity-related gene expression, gut microbiota, and metabolic pathways in mice. Microbiol. Spectr. 10, e0165122. doi: 10.1128/spectrum.01651-22 36321893 PMC9769980

[B31] Xiao H. W. Cui M. Li Y. Dong J. L. Zhang S. Q. Zhu C. C.. (2020). Gut microbiota-derived indole 3-propionic acid protects against radiation toxicity via retaining acyl-CoA-binding protein. Microbiome 8, 69. doi: 10.1186/s40168-020-00845-6 32434586 PMC7241002

[B33] Zhang T. Lu G. Zhao Z. Liu Y. Shen Q. Li P.. (2020). Washed microbiota transplantation vs. manual fecal microbiota transplantation: clinical findings, animal studies and *in vitro* screening. Protein Cell. 11, 251–266. doi: 10.1007/s13238-019-00684-8 31919742 PMC7093410

[B32] Zhang D. Zhong D. Ouyang J. He J. Qi Y. Chen W.. (2022). Microalgae-based oral microcarriers for gut microbiota homeostasis and intestinal protection in cancer radiotherapy. Nat. Commun. 13, 1413. doi: 10.1038/s41467-022-28744-4 35301299 PMC8931093

[B34] Zheng P. Zeng B. Zhou C. Liu M. Fang Z. Xu X.. (2016). Gut microbiome remodeling induces depressive-like behaviors through a pathway mediated by the host’s metabolism. Mol. Psychiatry 21, 786–796. doi: 10.1038/mp.2016.44 27067014

