# Supplementary material for: Fecal bacteria-free filtrate transplantation is proved as an effective way for the recovery of radiation-induced individuals in mice
Source: Front Cell Infect Microbiol. 2024 Jan 31;13:1343752. doi: 10.3389/fcimb.2023.1343752 (PMC10864540; doi:10.3389/fcimb.2023.1343752)
Supplement: Supplementary file 1 [file DataSheet_1.docx]

**Additional file 1**

**Preparation of fecal bacteria-free filtrate**

Specifically, the pooled stool material was resuspended by vortexing in sterile saline (10% weight/volume ratio), homogenized for 2 min, and subsequently centrifuged at 2500 g and 4℃ for 10 min to pellet undigested food debris and other large particles. The resulting solution was passed twice through a 0.45-μm pore size filter (Millipore) for the removal of bacterial cells, followed by depletion of bacteriome using a 0.22-μm diameter filter (Millipore). The concentration of resulting viral filtrate was determined using SYBR Gold method and fluorescence microscopy.

**Reads-based phylogenetic annotation**

Raw sequence reads underwent quality trimming using Trimmomatic (http://www.usadellab.org/cms/uploads/supplementary/Trimmomatic) to remove adaptor contaminants and low quality reads (Bolger et al., 2014). Reads through quality control were then mapped to the mouse genome (NCBI) by BWA mem algorithm (parameters: -M -k 32 -t 16, http://bio-bwa.sourceforge.net/bwa.shtml). The reads removing host-genome contaminations and low-quality data were called as clean reads and used for furthur analysis. According to the default database downloaded from Broad Institute (min-score-identity = 0.90, identity margin = 0.02), taxonomy of the clean reads for each sample was measured through the PathSeq pipeline distributed in GATK v4.1.3 (https://github.com/usadellab/Trimmomatic) (Kostic et al., 2011). All reads were then classified into several phylogenetic levels of interest.

**References**

Bolger, A. M., M. Lohse, and B. Usadel. 2014. Trimmomatic: a flexible trimmer for Illumina sequence data. Bioinformatics 30(15):2114-2120.

Kostic, A. D., A. I. Ojesina, C. S. Pedamallu, J. Jung, R. G. Verhaak, G. Getz, and M. Meyerson. 2011. PathSeq: software to identify or discover microbes by deep sequencing of human tissue. Nat Biotechnol 29(5):393-396.

Supplementary table 1. Primer sets used for qPCR.

| Gene | Forward primer sequence (5’-3’) | Reverse primer sequence (5’-3’) |
| --- | --- | --- |
| ZO-1 | GATTTACCCGTCAGCCCTT | TCCGACATCATTTCCACCA |
| Muc-2 | TTCGTCACTTCATCAGCGG | GTCTTTGAGGAGGTGGGCA |
| Glut1 | GCTTCCTGCTCATCAATCG | TGGTGACCTTCTTCTCCCG |
| Pgk1 | ACCAGATAACAAACAACCA | CTCATAAGGACAACGGACT |
| IL-10 | ACAACATACTGCTAACCGA | TCTCACCCAGGGAATTCAA |
| Nrf2 | CTGGAGAACATTGTCGAGC | GAGCCGCCTTTTCAGTAGA |
| IL-8 | TTCATTCCTCTCAAACTCA | AAACAAATCATACTCCCAT |
| IL-1β | AGAAGATGGAAAAACGGTT | CTTGTGCTCTGCTTGTGAG |
| IL-18 | ATACCTGAAGAAAATGGAG | TTTGTCAACGAAGAGAACT |
| TNF-α | TCAACCTCCTCTCTGCCGT | CTGAGTTGGTCCCCCTTCT |
| TGF-β | GGACCTGGGTTGGAAGTGG | GGTTGTGTTGGTTGTAGAG |
| PDGF-c | TGTATGGGAAAAAAAGCAA | CCCGTATGGACACTGAGAA |
| GAPDH | CCTTCCGTGTTCCTACCCC | GCCCAAGATGCCCTTCAGT |


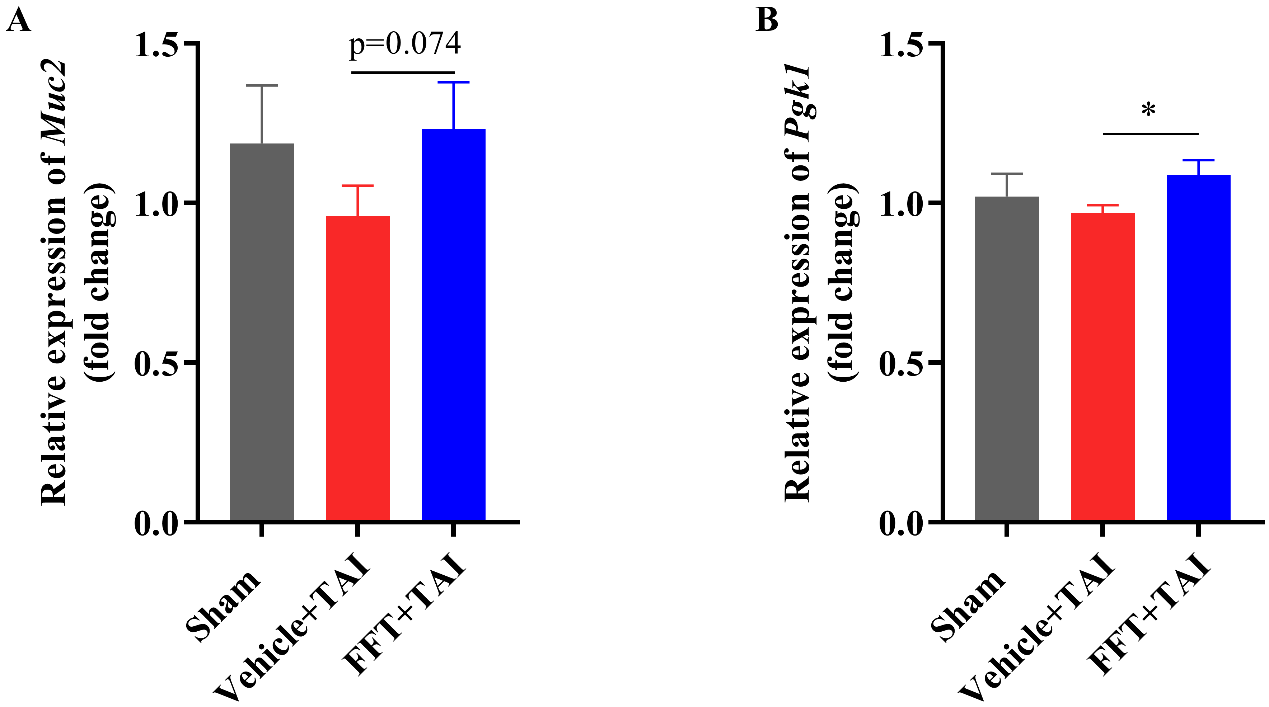


Figure S1. Relative mRNA expression level of the genes of interest in recipient mice. (A) Relative mRNA expression level of *Muc2*. (B) Relative mRNA expression level of *Pgk1*. ^*^*P* < 0.05. Data are reported as mean ± SEM.


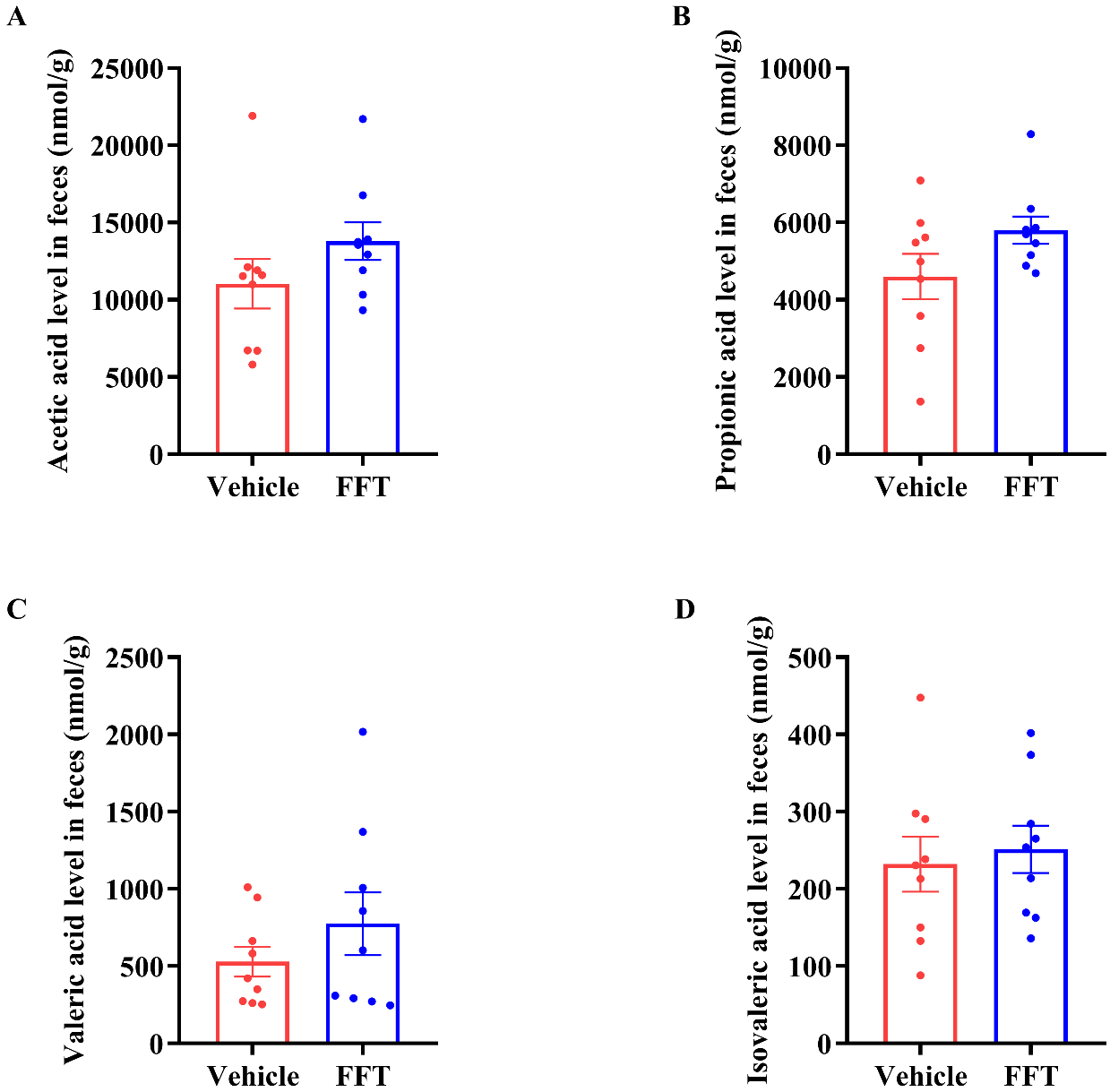


Figure S2. SCFAs levels in feces of recipient mice. (A) Acetic acid level in the feces of mice treated with FFT or vehicle. (B) Propionic acid level in the feces of mice treated with FFT or vehicle. (C) Valeric acid level in the feces of mice treated with FFT or vehicle. (D) Isovaleric acid level in the feces of mice treated with FFT or vehicle. Data are reported as mean ± SEM. Each symbol represents a mouse.

Graphical abstract:


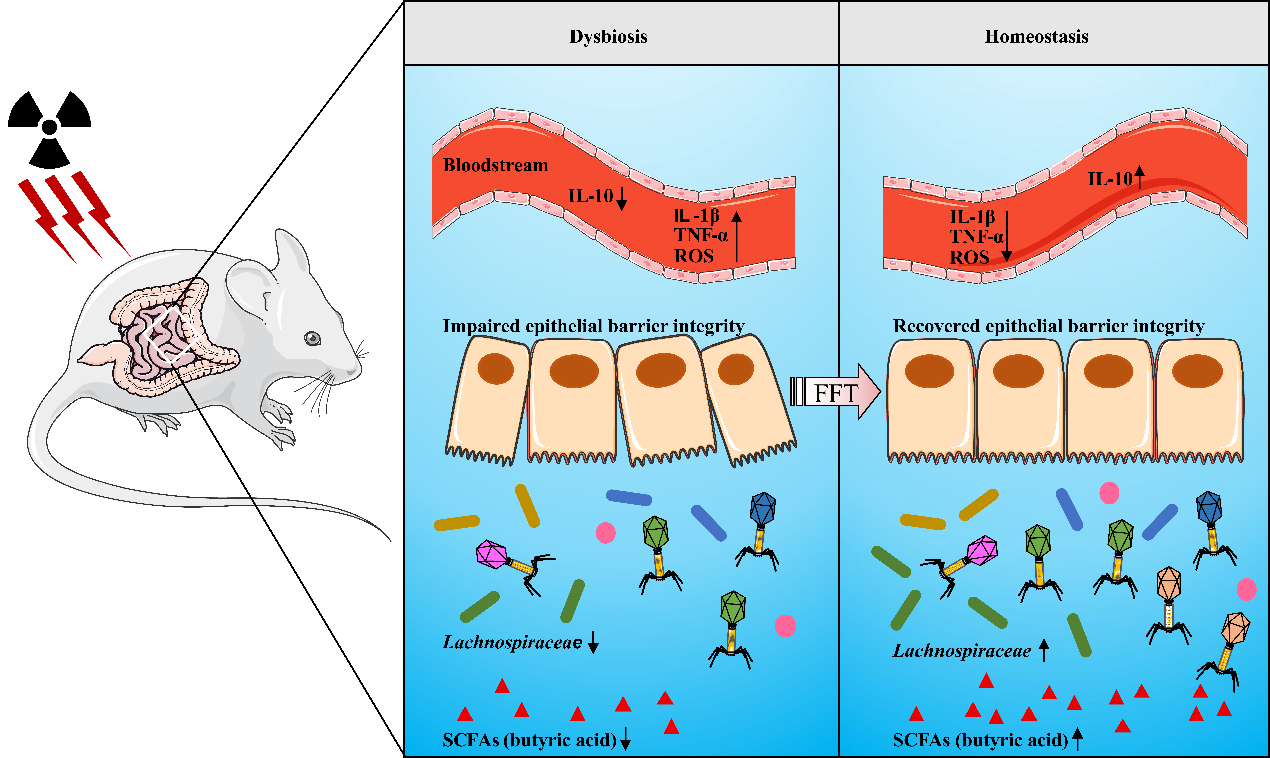


Radioprotective role mediated by FFT. FFT mitigates radiation caused dysbiosis by modulating intestinal barrier integrity, inflammatory response, gut microbiota, and host metabolism.
